# Public interest and engagement in care for brain health in Slovenia: the role of education

**DOI:** 10.3389/fpubh.2025.1490846

**Published:** 2025-03-12

**Authors:** Matej Perovnik, Hana Kos, Gaj Vidmar, Sara Fabjan, Hana Hawlina, Nastja Tomat, Dolores Trol, Mara Bresjanac

**Affiliations:** ^1^Slovenian Neuroscience Association – SiNAPSA, Ljubljana, Slovenia; ^2^Department of Neurology, University Medical Centre Ljubljana, Ljubljana, Slovenia; ^3^Faculty of Medicine, University of Ljubljana, Ljubljana, Slovenia; ^4^University Rehabilitation Institute of the Republic of Slovenia, Ljubljana, Slovenia; ^5^FAMNIT, University of Primorska, Koper, Slovenia; ^6^Faculty of Health Sciences, University of Primorska, Koper, Slovenia; ^7^The Institute of Criminology at the Faculty of Law, Ljubljana, Slovenia; ^8^Faculty of Arts, University of Ljubljana, Ljubljana, Slovenia; ^9^Ministry of Health of the Republic of Slovenia, Ljubljana, Slovenia

**Keywords:** brain health, brain disorders, disease prevention, public engagement, health literacy, knowledge gap

## Abstract

**Background and objective:**

Knowledge gap theory posits that individuals with better education have better opportunities to obtain, understand and utilise available information. In a health-related context, this insight could lead to a more effective disease prevention. The aim of our study was to test the hypothesis that knowledge gap underlies differences in behaviour aimed at maintaining brain health.

**Methods:**

We conducted an online survey investigating Slovenian public view on brain, brain research, and science-based recommendations for brain health. The survey was filled out by a total of 2,568 respondents, of whom 2,450 completed it in full. They were divided into two subgroups based on their self-reported brain-related education, i.e., the lay subgroup (*n* = 1,012) and a topically educated group (*n* = 1,438), i.e., the educated subgroup. Among the latter, 728 participants reported to have a Bachelor’s degree or higher education. We analysed the views of this sample subgroup on brain, neuroscience, and science-based brain health recommendations, and compared them with age- and education-matched lay subgroup (*n* = 565) from the same survey.

**Results:**

Educated individuals showed greater awareness and adherence to science-based recommendations compared to the lay respondents, specifically in the perceived importance of following a healthy diet, exercising, ensuring time for rest, relaxation and maintenance of social contacts, acquiring new knowledge and skills and using supplements that are considered to improve mental abilities (all *p* < 0.005), but not in the perceived importance of getting enough sleep, avoiding drugs and alcohol or injuries and performing mentally-challenging activities or cognitive training (all *p* > 0.10). Educated individuals more frequently reported following a healthy diet, engaging in physical activity and socialising, acquiring new knowledge and skills, performing mental challenges and cognitive training, and using supplements (all *p* < 0.005), but not getting sufficient sleep, avoiding drugs, alcohol or injury, or ensuring time for rest and relaxation (all *p* > 0.08). A larger proportion of lay than educated participants (32 and 17%, respectively) identified lack of information as a reason for not engaging in healthy practices (*p* < 0.001). Educated participants outperformed lay individuals in identifying diseases amenable to lifestyle modification.

**Conclusion:**

Understanding the differences in brain health perceptions between educated and lay individuals is crucial for developing effective public health strategies. Our results highlight a substantial knowledge gap in the Slovenian population and the need for targeted educational interventions that account for varying degrees of knowledge in different population segments which could lead to better adherence to healthy lifestyle practices.

## 1 Introduction

Even though “brain health” is becoming an increasingly popular term among lay-persons, health experts and researchers, there is still no clear or universally accepted definition (1). The WHO defines brain health as the state of brain functioning across multifaceted domains, allowing a person to realise their full potential (2). Similarly, the World Federation of Neurology characterises brain health as the capacity for communication, decision-making, problem-solving, and leading a productive life (3). Adding to this, the American Heart Association defines brain health as the absence of any brain disease and preservation of neuronal function to meet the demands of everyday life with capacity to function adaptively in one’s environment (4). Chen et al. (1) also posit that brain health represents a complex, evolving state across cognitive, emotional, and motor domains, supported by physiological functions, and can be quantified objectively as well as experienced subjectively. These definitions collectively underscore that brain health is not a static state but a dynamic process influencing a wide range of human functioning and wellbeing, measurable and improvable throughout the lifespan.

A comprehensive account of brain health needs to incorporate a notion of brain disorders, which are defined by the WHO as conditions resulting from disturbances in brain development, structural brain damage, and/or impaired brain function (2). They include both neurological and psychiatric disorders, which are classified within the same foundational framework due to their common neuroanatomical substrate – the brain. Despite being historically separated, neurology and psychiatry share many diagnostic and treatment methodologies. Mental disorders, such as depression and schizophrenia, and neurological disorders, such as epilepsy and dementia, are thus often considered as belonging to a single group of neuropsychiatric disorders or disorders of the brain (5). Reflecting the breadth of these definitions, our study adopts the widest conceptualization of brain disorders, aiming to explore their impact on the multifaceted nature of brain health.

Brain disorders are widely prevalent, resulting in significant short- and long-term disabilities, and impose substantial emotional, financial, and social costs on patients and their social circles (5). In 2016, neurological disorders were globally the leading cause of disability-adjusted life years [DALYs - the sum of years of life lost (YLLs) and years lived with disability (YLDs)] at 276 million and the second leading cause of death at 9 million, with an overall increase in absolute numbers but a decrease in age-standardised rates since 1990, except for decreases in tetanus, meningitis, and encephalitis (6). Stroke, migraine, Alzheimer disease, and meningitis were the top contributors to this burden (6). In 2019, mental disorders accounted for 125.3 million DALYs globally, ranking as the 7th leading cause, a significant increase from 80.8 million DALYs and the 13th position in 1990 (7). They were also the second leading cause of YLDs worldwide in both years, predominantly due to depressive and anxiety disorders, and schizophrenia (7). In Slovenia, the economic burden of brain diseases in 2010 amounted to 2.425 billion EUR purchasing power parity (PPP), equivalent to 7% of the gross domestic product (8). The average annual cost *per capita* for all brain diseases was estimated at 1.185 EUR PPP, positioning Slovenia’s expenditures within the median range among European countries, where the average was estimated at 1.550 EUR PPP per capita, or a total of 798 billion EUR PPP (8).

As Slovenian—and European—population ages (9, 10), it is anticipated that the costs associated with neurological diseases will consequently rise (11). In order to reduce the future economic and health burdens, it is essential to focus on “healthy ageing,” which is defined by the WHO as the capacity to develop and maintain the functional abilities that foster wellbeing into older age, emphasising that the absence of disease is not mandatory for this process (12). This concept includes the ability of individuals to keep their independence, maintain relationships, and contribute to society (12).

Reinforcing the importance of this approach, a 2003 study (13) revealed that older adult individuals in better health at age 70 not only have a longer life expectancy compared to those in poorer health, but also do not incur higher cumulative healthcare expenditures despite their extended lifespan. Supporting this, a 2023 study from Norway and Denmark (14) demonstrated that longer life expectancy does not necessarily lead to higher health care costs, especially among healthier older adult populations (14). The authors found that as life expectancy increases, health care spending patterns may resemble those of younger individuals, suggesting that end-of-life costs may not escalate as previously expected (14). These findings underscore the importance of promoting better health practices earlier in life as a crucial strategy for managing the future economic impacts of an ageing population, ensuring that longer lives do not necessarily translate to proportionately higher medical costs (13, 14).

In addition to vast social and financial costs, disorders of the brain also place great emotional burden on patients and their caregivers and affect their quality of life. A 2016 survey (15) revealed that individuals over the age of 50 predominantly fear Alzheimer disease and cancer. Consistently, a 2018 survey (16) showed that Malaysian residents aged 40 and older feared cancer, Alzheimer disease, and heart disease, with greater concern about becoming a burden to their family and the financial impact of their illness than the fear of dying. Numerous studies report findings about the psychological impact of various brain disorders on patients and their care-givers, such as reported high psychological burden, symptoms of depression (17) and reduced quality of life (18).

Emphasising healthy ageing as a crucial aspect of public health requires a better understanding and more effective promotion of healthy lifestyle practices. A 2021 review (19) suggested that patient education not only significantly improves health outcomes and enhances medical treatment but is also cost-efficient. Its effectiveness is pronounced across various health conditions, for example diabetes, circulatory system diseases, or post-surgical recovery, owing to well-established, tailored instructional methods that focus on lifestyle changes (19). A 2003 study, for example, highlighted that achievements in HIV prevention underscore the efficacy of community-based health promotion, emphasising the importance of community involvement and setting these initiatives apart from broader efforts (20). Likewise, a 2011 study (21) illustrated that education sessions significantly improved health behaviours and medication adherence in arterial hypertension patients, evidenced by notable blood pressure reductions. However, the variability in effectiveness indicates the need for detailed analysis to fully understand and optimise patient education interventions (19).

The article from Think Brain Health Global (22) emphasised the importance of primary prevention and early intervention in neurodegenerative diseases, advocating for a unified approach that spreads public awareness of modifiable risk factors, integrates health-promoting behaviours through supportive policies, and develops environments that encourage healthy lifestyles. It calls for healthcare professionals to motivate the public, enhances research to optimise disease management, and stresses the need for researchers and policymakers to collaborate in implementing effective public health strategies (22).

As suggested by several global initiatives (23–25), there is a need to promote attitudes and behaviours that contribute to brain health, reduce modifiable risk factors for the onset of brain disorders, and contribute to the wellbeing and quality of life of patients and their caregivers. In 2021, we reported on the first online survey investigating the Slovenian public’s knowledge and adherence to brain health recommendations. At the time, we divided the survey respondents’ sample into two subgroups based on the self-reported brain-related education. The lay subgroup was analysed first, and key findings were reported (26). The remaining respondents represented a topically educated group. Here we presented the views of this survey sample subgroup on brain, neuroscience and science-based brain health recommendations, and compared them with the lay public views and reported behaviours. The overarching aim of this study was to test the hypothesis that knowledge gap underlies differences in behaviour aimed at maintaining brain health.

## 2 Methods

### 2.1 Procedure and participants

In August 2017, we conducted a survey under the auspices of the project “Z možgani za možgane” (Aim for the Brain) that was filled out in part or completely by 2,568 participants. Only the data from 2,450 individuals who filled the survey in full was analysed.

The survey questions were based on 200 structured interviews conducted between March and July 2017 with a wide range of individuals representing diverse age, gender, education and employment status across Slovenia (*details not shown*).

The respondents were divided into two separate subgroups based on their self-reported brain-related education. Respondents (*n* = 1,012) who had not received formal education about the brain and did not professionally rely on brain-related knowledge were considered to represent the lay public and key findings about their attitudes and behaviours were reported previously (26). The remaining respondents (*n* = 1,483) were considered to have some, albeit varying degrees of expertise in brain-related subjects and are henceforth addressed as an educated group. The participants (*n* = 710) in this group who had a lower level of education than a Bachelor’s degree or equivalent were excluded from the analysis to improve the homogeneity of our sample.

This was a cross-sectional observational study and the survey methodology is described in more detail in (26). Briefly, participants were recruited through social media channels like the project’s Facebook page, mailing lists of the Slovenian Neuroscience Association (SiNAPSA), student organisations (e.g., Slovenian Psychology Students Association), and partner websites (e.g., http://umni.si/). The online data collection took place over 3 weeks. The only inclusion criterion was knowledge of Slovene. Participants were given the option to enter a random draw for a selection of practical, health-friendly prizes. The contact information was stored separately from their answers. Participants were asked to: (1) assess their knowledge of the brain and the significance of brain health (using a five-point scale); (2) indicate the importance and their involvement in activities enhancing brain health (choosing from a list of options); and (3) describe their experiences with sources of brain-related information (choosing from a list of options). Basic demographic information was also collected. The entire survey (in Slovene) is available at: https://www.1ka.si/a/280638 and the English translation at: https://www.frontiersin.org/journals/public-health/articles/10.3389/fpubh.2021.690421/full#supplementary-material.

### 2.2 Statistical analysis

Student’s independent sample *t*-test and *chi*-square test were used to examine the demographic differences between the two groups. We applied propensity score (PS) regression, i. e. PS estimation followed by regression models with group and PS as predictors, whereby PS was estimated using Firth (bias-corrected) logistic regression based on potential confounders (age, gender, educational level, region, diagnosis of neurological or psychiatric disorder of the respondent and of their family member/s). Responses on five-point Likert-type scales (1 = not at all to 5 = very much for importance ratings and 1 = never to 5 = every day for compliance ratings) and number of brain conditions deemed to be preventable were modelled using multiple linear regression. Lack of brain health-protective practices due to lack of information was modelled using multiple logistic regression. To illustrate the difference between the groups in the opinion on preventability of brain diseases, an unadjusted univariate comparison was also conducted using *t*-test and *chi*-square test. IBM SPSS Statistics 23 (IBM Corp. Armonk, NY, United States) software was used for data analyses. RStudio 2024.04.2 + 764, R version 4.4.1 (R Foundation for Statistical Computing, Vienna, Austria) was used for plotting. The results were considered statistically significant at *p* < 0.05 (two-tailed).

## 3 Results

**Figure 1:**
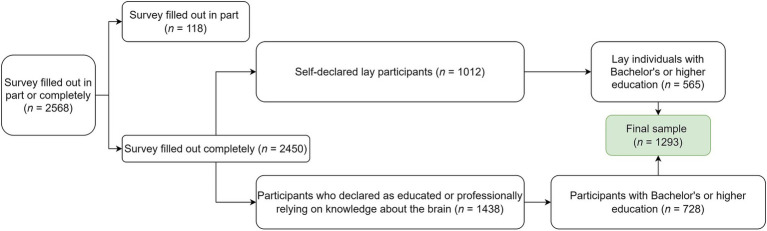
Flowchart of the selection procedure.

We present results from a subgroup of 728 participants who declared themselves to be educated or professionally reliant on knowledge about the brain and to hold a Bachelor’s degree or higher, and a group of age- and education-matched individuals (*n* = 565) representing the lay population from the same survey. Flowchart of the selection procedure is shown in Figure 1. The groups differed in the sex distribution, with a higher proportion of women in the educated (80.2%) than the lay group (73.3%), *χ*^2^(1) = 8.714, *p* = 0.003. The educated group was statistically significantly older on average [mean (M) = 39.1, standard deviation (*SD*) = 13.8] than the lay group [*M* = 37.1, *SD* = 12.0; *t*(1274.4) = 2.885, *p* = 0.004]. The two groups also differed in home region distribution [*χ*^2^(11) = 28.496, *p* = 0.003], with central Slovenia region being most common and Littoral–Inner Carniola region the least common overall. The two groups did not differ statistically significantly in education level [*χ*^2^(3) = 5.724, *p* = 0.126], respondents’ neurological or psychiatric disease diagnosis [*χ*^2^(1) = 0.82, *p* = 0.775] or diagnosis of neurological or psychiatric disease among respondents’ family members [*χ*^2^(1) = 0.033, *p* = 0.856] (Table 1).

**Table 1:** Demographic data.

Variable	Educated	Lay	*p*-value
*N*	728	565	
Age (Y)	39.1 (13.8)	37.1 (12.0)	0.004
Sex (f, %)	80.2	73.3	0.003
Education level (Y)	7.8 (0.9)	7.7 (0.9)	0.126
Diagnosis of a neurological or psychiatric disorder (y, %)	15.5	16.1	0.775
Relative diagnosed with a neurological or psychiatric disorder (y, %)	63.3	62.8	0.56

Y, years; y, yes; f, female; numerical variables are reported as mean (SD).

**Figure 2:**
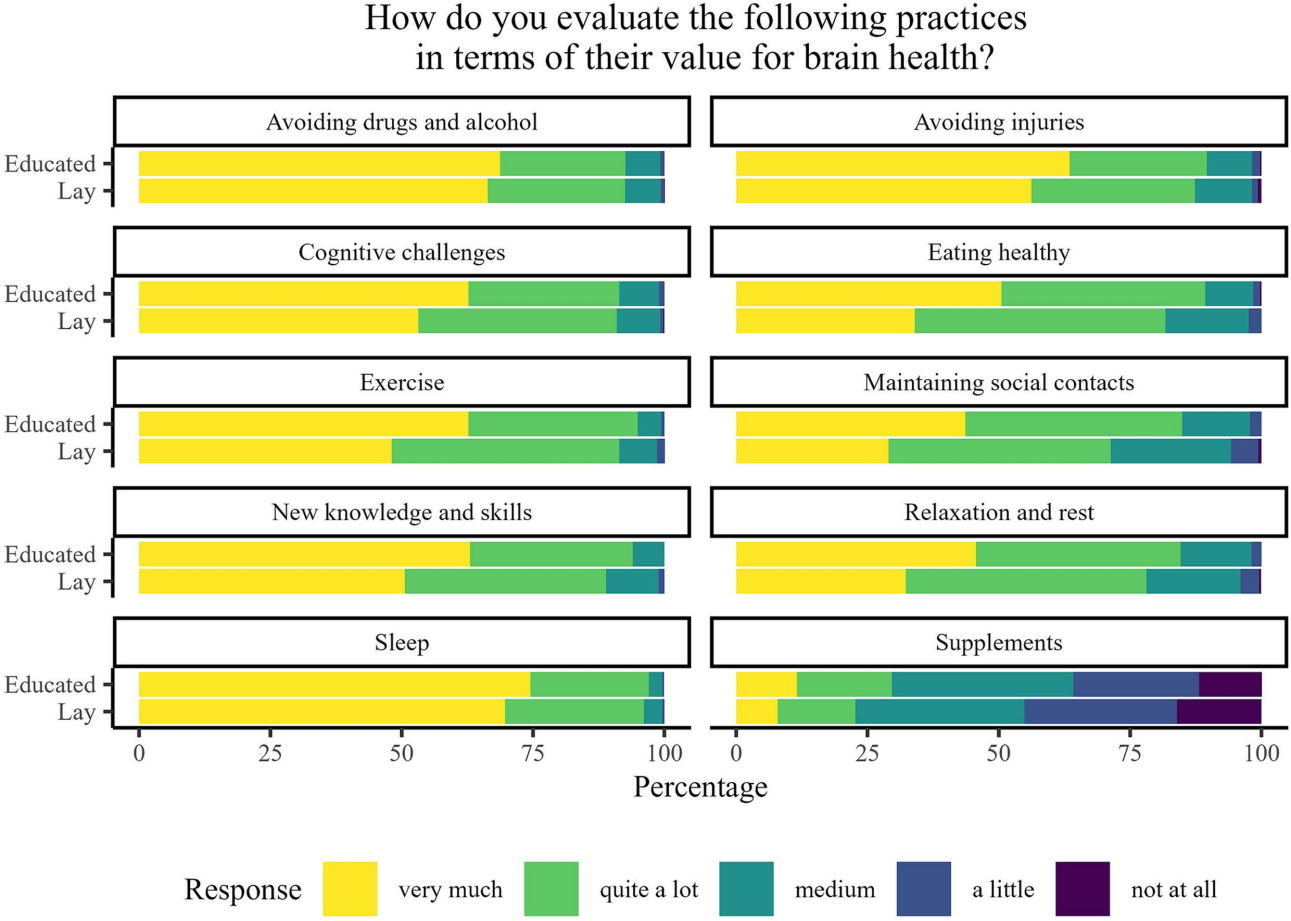
Percentage of responses to the question “How do you evaluate the following practices in terms of their value for brain health?” stratified by participants’ brain-related education.

**Figure 3:**
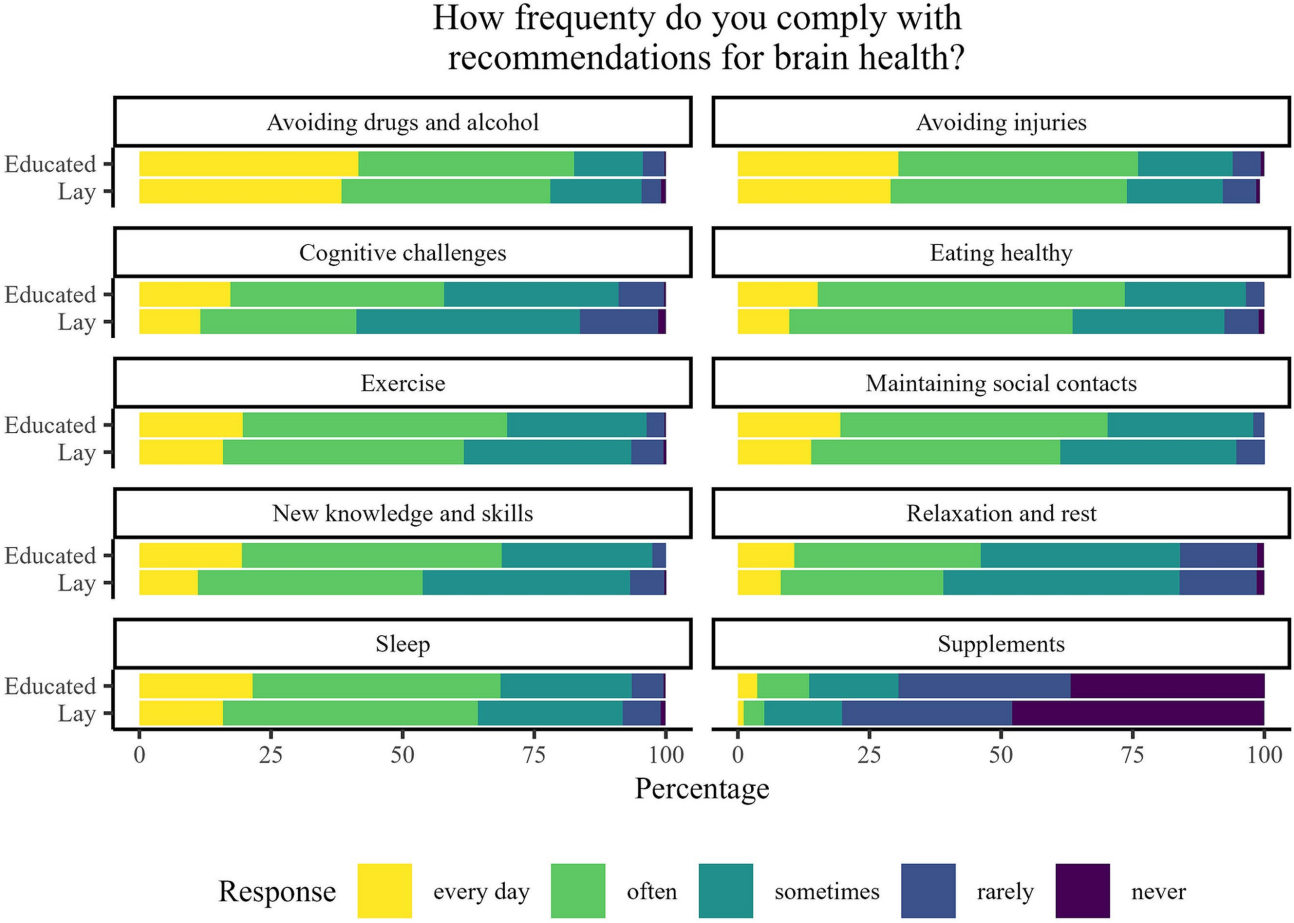
Percentage of responses to the question “How frequently do you comply with recommendations for brain health?” stratified by participants’ brain-related education.

The majority of participants from both educated and lay groups considered all of the practices, except supplement intake, to be *very* or *moderately important* for brain health. However, on average, the educated group rated all of the practices as more important for brain health than the lay group (Figure 2). Similar differences among lay and educated public are seen in Figure 3, as the educated group reported that they more often comply with recommendations for brain health in all of the listed practices.

The PS-adjusted linear-regression-based comparisons are summarised in Table 2. All models were statistically significant. A statistically significant difference between the educated and lay public was observed regarding 14 of the 22 analysed questions. In all those questions, the average rating of the educated public was higher. Statistically significant differences in perceived importance between the educated and lay groups were found in the areas of overall importance of brain health, following a healthy diet, exercising, ensuring time for rest, relaxation and maintenance of social contacts, acquiring new knowledge and skills and using supplements that are considered to improve mental abilities. Similarly, educated participants reported, on average, statistically significant more frequent engagement in maintaining a healthy diet, exercising, socialising, acquiring new knowledge and skills, performing mental challenges and cognitive training, and using supplements, as compared with lay participants.

**Table 2:** Summary of propensity-score adjusted linear regression models for comparing the survey responses between educated and lay public.

Question		Lay vs. educated	
	*p* (Model)	ß*	*p*
How important is brain health to you?	< 0.001	−0.103	< 0.001
Importance for brain health—eating healthy	< 0.001	−0.145	< 0.001
Importance for brain health—exercise	< 0.001	−0.124	< 0.001
Importance for brain health—enough sleep	0.045	−0.044	0.128
Importance for brain health—avoid drugs and alcohol	< 0.001	0.022	0.450
Importance for brain health—avoiding injury	< 0.001	−0.047	0.104
Importance for brain health—relaxation and rest	< 0.001	−0.111	< 0.001
Importance for brain health—maintaining social contacts	< 0.001	−0.175	< 0.001
Importance for brain health—acquiring new knowledge and skills	< 0.001	−0.130	< 0.001
Importance for brain health—mental challenges and cognitive training	< 0.001	−0.043	0.136
Importance for brain health—using supplements	< 0.001	−0.083	0.004
Comply with recommendations for brain health—eating healthy	< 0.001	−0.105	< 0.001
Comply with recommendations for brain health—exercise	0.001	−0.084	0.004
Comply with recommendations for brain health—enough sleep	< 0.001	−0.052	0.075
Comply with recommendations for brain health—avoid drugs and alcohol	< 0.001	−0.003	0.907
Comply with recommendations for brain health—avoiding injury	< 0.001	0.002	0.931
Comply with recommendations for brain health—relaxation and rest	0.037	0.046	0.116
Comply with recommendations for brain health—maintaining social contacts	< 0.001	−0.121	< 0.001
Comply with recommendations for brain health—acquiring new knowledge and skills	< 0.001	−0.176	< 0.001
Comply with recommendations for brain health—mental challenges and cognitive training	< 0.001	−0.154	< 0.001
Comply with recommendations for brain health—using supplements	< 0.001	−0.147	< 0.001
Decreasing the probability—number of diseases	0.001	−0.101	< 0.001

*Standardised regression coefficient; negative value indicates higher average in the educated public group, positive in the lay public group.

It should be noted that the results of the PS-adjusted regression models were mainly consistent with unadjusted comparisons. Among importance ratings, the exceptions were the differences in avoiding drugs and alcohol, avoiding injury, and engaging in mental challenges and cognitive training, which were only statistically significant in unadjusted comparisons (*data not shown*). Similarly, regarding compliance, statistical differences between the two groups were only observed in obtaining sufficient sleep, avoiding drugs, alcohol and injury, and ensuring adequate rest and relaxation in unadjusted comparisons (*data not shown*).

**Figure 4:**
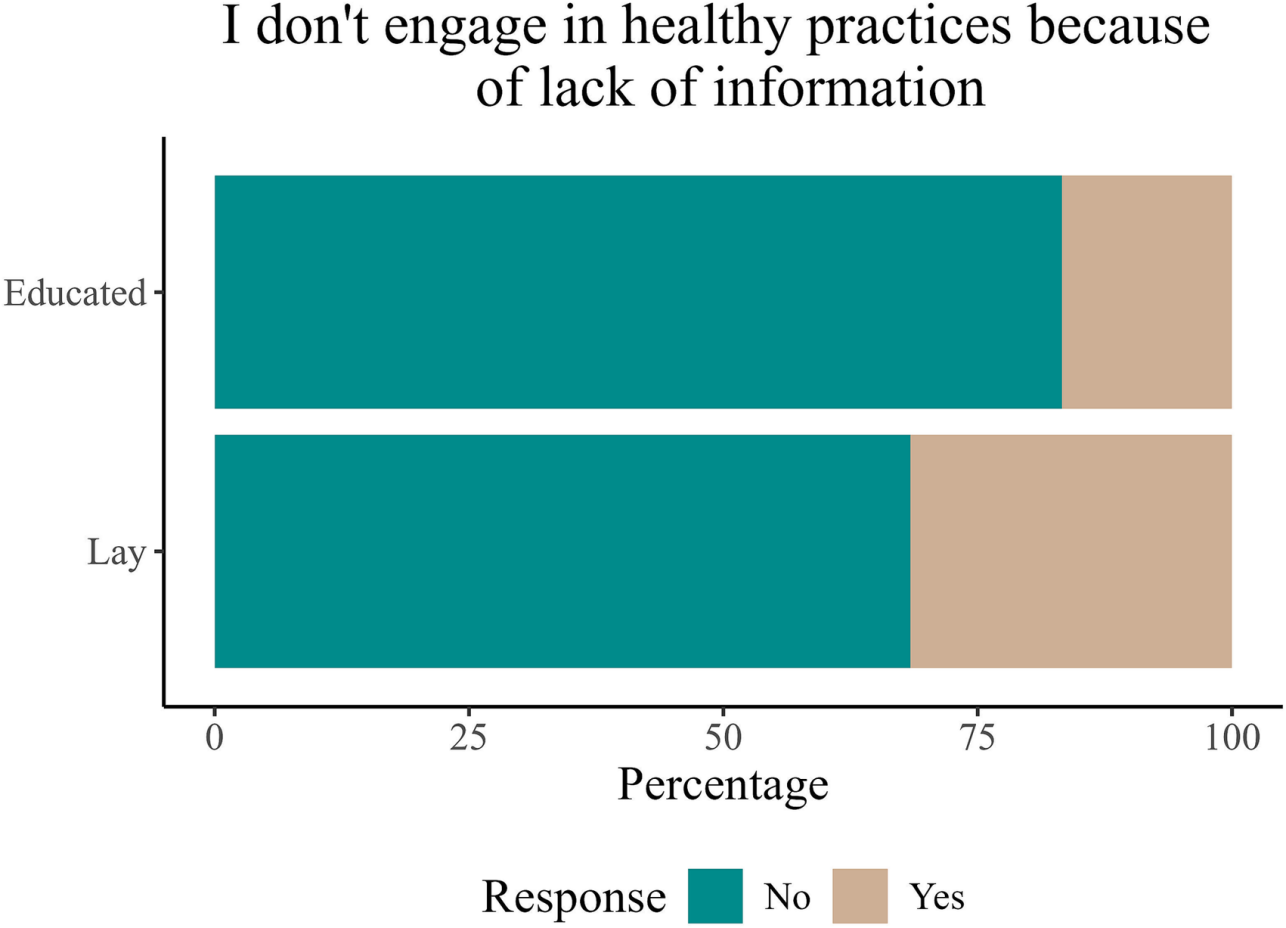
Percentage of responses to the question “I do not engage in healthy practices because of lack of information” stratified by participants’ brain-related education.

A larger proportion of lay than educated participants (32 and 17%, respectively) identified lack of information as a reason for not engaging in healthy practices [*χ*^2^(1) = 39.495, *p* < 0.001, Figure 4]. In addition, the PS-adjusted logistic regression model indicated that the educated public group responded statistically significantly less frequently that they did not engage in healthy practices because of lack of information (model *p* < 0.001, *p* for group comparison <0.001, estimated odds ratio educated vs. lay = 0.46, 95% confidence interval 0.35–0.61). However, the majority of both groups still believed they had enough information about what contributes to brain health, Figure 4.

Participants were asked to state whether they thought that the probability of the onset of listed neurological and psychiatric disorders could be decreased by a healthy lifestyle and taking care of the brain. The difference regarding the number of preventable brain diseases/disorders was confirmed by the univariate comparison, which showed that the educated group identified on average more diseases (*M* = 5.98, *SD* = 2.83) as having modifiable risk factors than the lay public group [*M* = 5.47, *SD* = 2.62; *t*(1251.8) = 3.398, *p* < 0.001].

**Figure 5:**
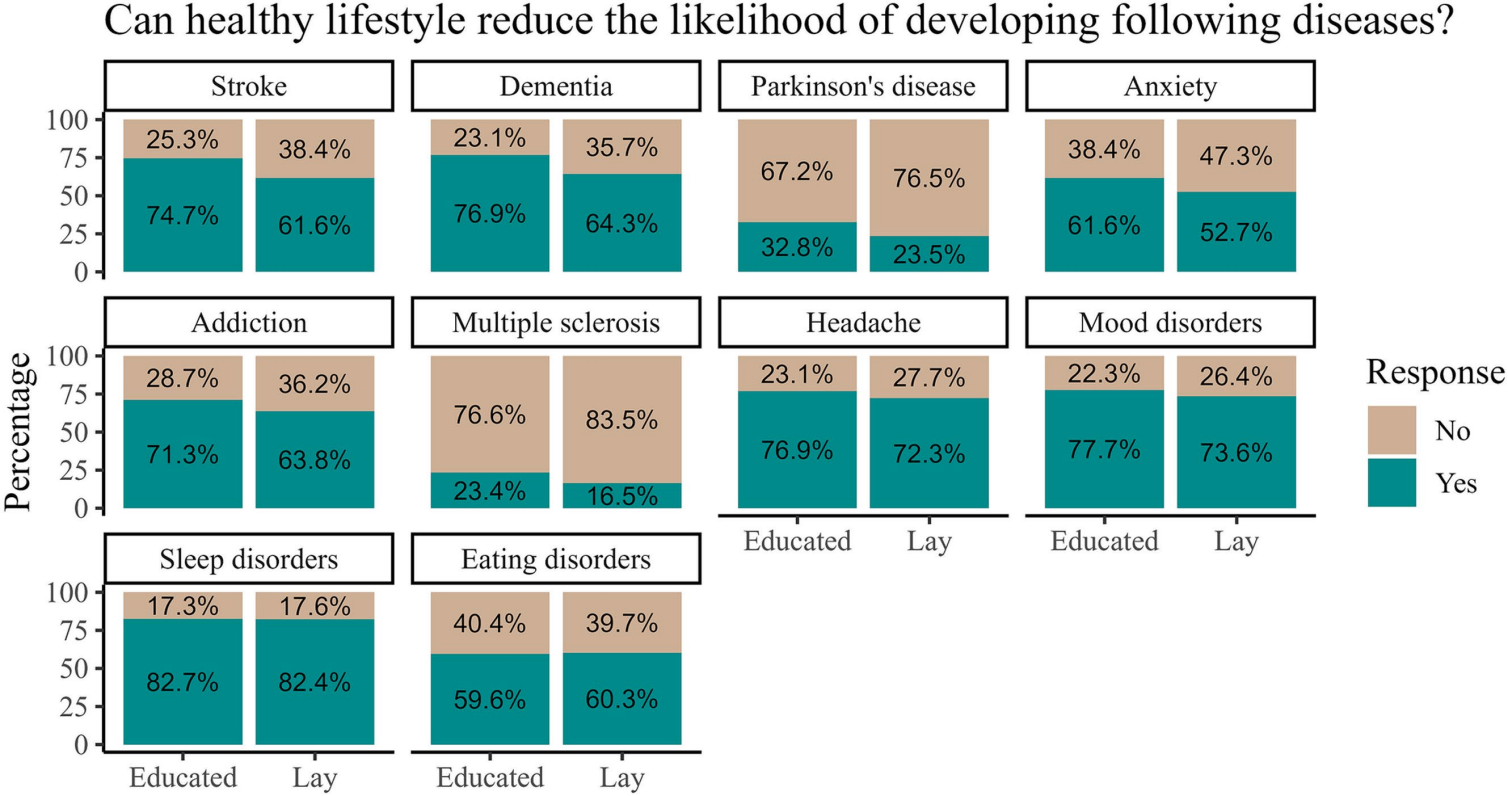
Proportion of participants that identified specific diseases in which we can reduce the likelihood of appearance by healthy lifestyle stratified by participants’ brain-related education.

More than half of the participants in both groups believed that the likelihood of the onset of all the disorders except for multiple sclerosis (MS) and Parkinson disease (PD) could be influenced by a healthy lifestyle, Figure 5. The percentage of participants in both groups who answered that lifestyle may influence the onset of the disease was the largest for sleep disorders, followed by mood disorders, headache, and dementia. A significantly higher proportion from the educated group responded that healthy lifestyle reduces the likelihood of stroke [*χ*^2^(1) = 25.64, *p* < 0.001], dementia [*χ*^2^(1) = 25.02, *p* < 0.001], PD [*χ*^2^(1) = 13.66, *p* = 0.0002], anxiety [*χ*^2^(1) = 10.08, *p* = 0.001], addiction [*χ*^2^(1) = 8.38, *p* = 0.004] and MS [*χ*^2^ (1) = 9.32, *p* = 0.002]. The unadjusted differences between the groups were not statistically significant for headache [*χ*^2^(1) = 3.75, *p* = 0.05], mood disorders [*χ*^2^(1) = 2.95, *p* = 0.09], sleep disorders [*χ*^2^(1) = 0.01, *p* = 0.92] and eating disorders [*χ*^2^(1) = 0.07, *p* = 0.79].

## 4 Discussion

In this paper we present the results from the first Slovenian national survey comparing knowledge of and engagement in brain health-related practices between self-reported lay and educated individuals (the latter having self-described as having attained or being in the process of attaining formal education involving systematic learning about structure and functioning of the brain, or professionally relying on their knowledge about brain structure and function, who had attained at least a Bachelor’s degree). The results indicate that educated individuals on average not only had greater awareness and implemented these practices more frequently, but also better recognised the impact of a healthy lifestyle on disease occurrence.

### 4.1 Healthy practices

Most participants in both groups identified sleep as a very important practice, followed by avoiding drugs, alcohol, and injuries. There were no statistically significant differences in responses between the groups for any of these questions. While the negative effects of drugs and alcohol on brain health are well established (27, 28), the positive effect of sleep has also gained significant traction in recent decades (29, 30). Therefore, it is not surprising that the majority of participants in both groups perceived sleep as very important for brain health, as they have received not only significant scientific attention but also popular scientific promotion.

The survey asked about two different kinds of purposeful mental engagement: (i) predominantly domain specific cognitive training, and (ii) a general activity devoted to learning new knowledge and skills. The proportion of participants that rated the activity as very important was slightly larger for cognitive training than for acquiring new knowledge and skills, with the educated group practising both activities more frequently. Computerised cognitive training (CCT) is often marketed as an effective mean for cognitive decline prevention. Studies show that cognitively active seniors are more likely to maintain their cognitive abilities compared to cognitive inactive peers (31), and CCT was shown to have positive effects on tests of cognition in individuals with mild cognitive impairment (32). However, the efficacy of CCT on real-world cognitive challenges remains to be demonstrated (33). Acquiring new knowledge and skills induces significant structural and functional brain changes (34). Based on our survey, educated individuals placed greater importance on and more frequently implemented these practices compared to lay individuals. Thus, increasing awareness and education in this area would be beneficial.

Educated individuals perceived healthy diet and physical activity as having a greater impact on brain health relative to the lay group, and they also more frequently reported engaging in both. Beneficial effects of physical activity have been demonstrated with respect to general health and wellbeing through its effects on cardiovascular and metabolic health, as well as in ameliorating symptoms of depression, anxiety and stress, and improving general cognitive performance (30, 35–37). Studies indicate that healthier nutrition is associated with a reduced risk of cognitive decline and neurodegenerative diseases (38, 39), while poor diet quality often coincides with neuropsychiatric disorders in which sedentary behaviours and poor sleep hygiene are also more common (30). In the latter group poor diet, lack of exercise and poor sleep hygiene can be both a consequence and a risk factor. Regardless of the directionality, promoting higher diet quality and regular physical activity is advisable to reduce the global burden of neuropsychiatric diseases (30, 35).

Respondents stated that maintaining social contacts is one of the less important categories and reported practising it less frequently. However, its importance was rated higher by the educated group, who reported that they strove to maintain social connections in their lives. Recently, especially with the COVID-19 pandemic, there has been increasing discussion about the importance of maintaining social contacts, as their lack can lead to loneliness (40), which is associated with higher morbidity and mortality, as well as increased incidence of mental disorders such as depression, anxiety, and psychotic disorders (41). Additionally, loneliness is a risk factor for the development of cognitive decline, including dementia due to Alzheimer disease (42). It most frequently affects the older adult, who represent the most vulnerable social group and the primary target for interventions aimed at reducing its deleterious effects on health and wellbeing (43).

Relaxation and rest were also thought to be of relatively less importance and were less frequently practised. Educated individuals assigned leisure greater importance for brain health than lay respondents, but engagement levels did not differ between the groups. As the concept was not explicitly defined, respondents may have understood it in different ways (e.g., afternoon naps, yoga and meditation, regular breaks during working hours, etc.), and their understanding of it may have overlapped with other activities, such as maintaining social contacts. This may explain why the perceived importance and engagement levels were relatively low with respect to other healthy practices. Despite the arguably inadequate specificity, securing time for rest and relaxation has been shown to influence overall health, especially by reducing stress (44).

Taking supplements was perceived as the least important for brain health in both groups, as the number of educated or lay individuals, who rated the practice as *very or moderately* important, was the lowest among all the health-related practices. An even smaller proportion of participants in both groups reported taking them daily or often. Interestingly, their use was more prevalent among educated individuals. According to literature, the most popular dietary supplements among US adults in 2019 were vitamins, protein, calcium, omega-3 fatty acids, green tea, magnesium, probiotics, iron, and turmeric, with their collective popularity rising (45). The main motivations for using supplements are to enhance overall health and wellness or to compensate for nutrient deficiencies (46), hence not specifically the care for brain health. Nevertheless, the effectiveness of dietary supplements outside the actual deficiency states remains questionable at best. People who use dietary supplements tend to engage in a range of healthy behaviours, suggesting that supplement use is integrated into a broader healthy lifestyle approach (46) and educated individuals in our sample reported following a healthier lifestyle overall. Two systematic reviews, however, concluded that there is no evidence that supplement use is associated with a lower risk of mild cognitive impairment or dementia (47, 48). Our results could suggest that the educated individuals are more aware of the existence of supplements that are marketed to improve mental abilities, but are not aware of the lack of scientific evidence to support such claims. A topic that gained significant attention in the last decade is the research on the gut-brain axis and the beneficial effects of probiotics supplements. A study from 2021 found that probiotic supplements improved mental flexibility and alleviated stress in healthy older adult individuals (49) and preclinical studies have also shown that dietary antioxidant supplements can be effective in reducing oxidative stress. Nevertheless, the authors stress that it is generally better to obtain antioxidants from whole foods rather than supplements (50). Overall, even though some findings suggest potentially beneficial effects of certain supplements for brain health, the general state of evidence indicates their use primarily in addressing specific nutritional deficiencies.

As expected, the lack of information was a more significant reason for not engaging in healthy practices among the lay segment of our survey sample compared to the educated group. In our previous report (26), we found that the lack of information was the second most common reason for not following brain health recommendations. Therefore, the lay public represent an important target population for educational interventions aimed at increasing awareness of and engagement in lifestyle practices to promote brain health.

### 4.2 The respondents’ views on the possibility of disease prevention

While the importance and effects of various healthy practices have been thoroughly discussed, it is essential to consider how these lifestyle choices influence the occurrence of neurological diseases. In our survey, we included stroke, dementia, PD, anxiety, addiction, MS, headache, and mood, sleep and eating disorders. In the following paragraph, we discuss each disorder starting with the one that the most respondents listed as being modifiable.

Respondents of both groups and to a similar degree stated that a healthy lifestyle can effectively reduce the likelihood of developing sleep disorders. Healthy lifestyle practices were shown to significantly improve sleep disorders in as little as 4 weeks, with long-term benefits depending on the maintenance of these changes (51). The improvement was primarily ascribed to decreased BMI and increased exercise (51), as regular physical activity has been found to improve sleep quality (52).

The second condition that respondents deemed amenable to a healthy lifestyle was headaches. Here, the difference in responses between the groups was also minimal. Studies have repeatedly shown that an unhealthy lifestyle is associated with a higher incidence of headaches (53, 54). Additionally, research has demonstrated that maintaining a consistent healthy lifestyle is a crucial element in effective behavioural strategies for preventing migraines (55).

Similarly, there were no differences in views between the educated and the lay group concerning the occurrence of mood disorders. Mood disorders include conditions like depression and bipolar disorder and are common psychiatric disorders leading to increased morbidity and mortality (56). However, numerous studies suggest that a healthy lifestyle coincides with less frequent occurrence (57, 58). Furthermore, there is particularly strong evidence supporting the beneficial impact of physical exercise (59).

Following mood disorders is dementia, which engendered one of the largest differences in responses – in fact, the second largest difference overall between the groups. It has been shown that a healthy lifestyle can reduce or delay the occurrence of dementia (60, 61). Indeed, up to 45% of dementia cases could potentially be prevented or delayed by addressing modifiable risk factors, such as hearing loss or elevated blood pressure, indicating a high potential for prevention (62). Given the substantial gap in awareness regarding the impact of a healthy lifestyle on the incidence of dementia, where the lay public is significantly less aware of this association, there is a crucial need for progress in public education. As mentioned previously, dementia is among the most feared diseases (15, 16), generating high costs (63) and overall public health burden. Increasing awareness about its preventability could serve as a strong motivation for adopting a healthier lifestyle.

The condition for which we observed the greatest difference in responses between the educated and lay people was stroke. Because stroke is one of the leading contributors to DALYs globally (6), it is concerning that 25.3% of the educated group and as much as 38.4% of the lay group did not express that a healthy lifestyle can influence its occurrence. The 2016 INTERSTROKE study identified 10 modifiable risk factors collectively accounting for approximately 90% of the population attributable risk for stroke globally, highlighting the need for prevention strategies (64).

Addiction was the sixth most frequently recognised condition that respondents stated was amenable to a healthy lifestyle with a significant difference between the educated and the lay group. Several studies have established an inverse association between addiction and healthy lifestyle practices, showing that less adherent individuals had a higher risk of problematic drinking, food and smoking addiction relative to persons leading a healthier lifestyle (65, 66). Furthermore, studies have shown that physical activity can be beneficial for those with substance use disorders, as it can enhance the rate of abstinence among users (67). Moreover, physical activity could be effective as an adjunctive treatment for individuals dependent on opioids (68).

Eating disorders exhibited the smallest difference in responses between the educated and lay people in our survey. Eating disorders rank as the third most prevalent chronic illness among adolescent girls (69). Regarding the impact of a healthy lifestyle on the prevalence of eating disorders, a 2016 article emphasises the importance of maintaining a healthy lifestyle, especially through family-based approaches. However, it highlights the need to avoid terms like “healthy eating” and discussions about body weight, as these could inadvertently contribute to the development of eating disorders (60).

Anxiety ranked among the top three conditions where perceived impact of healthy lifestyle practices was lowest. However, it has been shown that unhealthy lifestyle is associated with anxiety (70) and a lifestyle modification is an effective way for reducing anxiety symptoms (71).

Less than a third of our respondents in either group stated that healthy lifestyle practices can reduce the risk for developing PD and MS. While many studies have investigated risk factors for developing PD, there is little conclusive evidence regarding how lifestyle affects its incidence (72). However, there is some suggestion that physical exercise can reduce the risk of developing the disease, improve symptoms (73, 74), and even decrease mortality (75). Additionally, a healthy diet has also been shown to be beneficial (75, 76). There is some evidence that following a healthy lifestyle is associated with a lower prevalence of MS (77) and has an overall beneficial impact on the quality of life of those affected with disease (78–80). Infection with the Epstein Barr virus (EBV) has been identified as a predisposition for most forms of multiple sclerosis (81, 82). Studies suggest that certain environmental and lifestyle factors, especially those impacting the immune system, could synergistically interact with the EBV, increasing the risk of developing MS (83, 84).

### 4.3 Strengths and limitations

Our study is the first to test the knowledge gap explanation for putative differences in understanding of and adherence to science-based recommendations that favour brain health in Slovenia. This report provides insights into the attitudes about brain health and engagement in brain-health related activities in a sample of Slovenian educated public and their comparison with the attitudes and behaviours of lay public. The findings could serve as a starting point for designing specific, tailored interventions for improving brain health awareness and engagement in activities that improve prevention of brain disorders. While the data collection took place in 2017 the findings remain relevant and a follow-up study was recently devised and implemented.

The main limitation of the study was that participants were categorised as educated or lay based on self-reported brain-related education and knowledge. As we explained in the survey, participants were asked to state if they have formal education which includes systematic learning about the structure and functioning of the brain, if they rely on their knowledge about the brain in the workplace or if they actively educate others about brain related subjects, or if none of those options applies to them. They were assigned to the lay or educated groups based on their answers. In future studies, it would be beneficial to define fixed criteria for topically educated public, such as having a formal education in medicine, psychology, or related fields, or having an occupation that requires engaging with brain-related subjects, such as a doctor, a neuroscientist or a lecturer in a relevant field, and assign participants to groups according to those criteria. We increased the homogeneity of the informed group by excluding all participants that self-reported attainment of or involvement in the process of attaining formal education which includes systematic learning about structure and functioning of the brain, but did not obtain at least a Bachelor’s degree. Online survey format dictates reliance on respondents’ reported characteristics, including education.

As we collected data online, we could not reach individuals without access to a computer or basic digital skills. As already stated in our previous work (26), it would be beneficial if future studies added a paper-and-pencil version of the survey. Furthermore, it would be interesting to explore participants’ understanding of how and why a specific activity is beneficial for brain health, or if they engage in activities primarily out of concern for brain health, for health in general or some other reason, and why they think that some disorders may be influenced by lifestyle and others cannot be. Understanding the reasoning and reasons underlying participants’ attitudes and beliefs about brain health may help us plan more targeted interventions, for example by addressing misinformation on a specific topic, or design tailored interventions to improve engagement in brain-health promoting activities.

## 5 Conclusion

Given the increasing prevalence of neurological disorders and the ageing population, integrating brain health education into broader health promotion efforts is not only timely but necessary. Such efforts could have a substantial impact on public health outcomes, including the potential to delay or prevent the onset of debilitating brain diseases such as dementia, stroke and depression (62, 85–87). As highlighted in our study, we identified a significant knowledge gap in brain health literacy between the educated and lay public, with the former group demonstrating greater awareness, adherence to science-based recommendations, and understanding of the impact of lifestyle choices on brain health.

Promoting brain-healthy behaviours through public education campaigns could be a cost-effective strategy to reduce the incidence of these conditions and improve quality of life for older adults (5, 22, 88). To this end, policymakers and healthcare providers should prioritise brain health education, ensuring that accurate, accessible information is available to all segments of the population (89, 90). Future research should explore the most effective methods for brain health education and measure the long-term impacts of such interventions on public health outcomes. By closing the knowledge gap identified in this study, we can foster a more informed, proactive population that is better equipped to maintain cognitive health and mitigate the risks associated with brain disorders.

## Data Availability

The raw data supporting the conclusions of this article will be made available by the authors, without undue reservation.
